# Artificial Inclusion Bodies for Clinical Development

**DOI:** 10.1002/advs.201902420

**Published:** 2019-11-27

**Authors:** Julieta M. Sánchez, Hèctor López‐Laguna, Patricia Álamo, Naroa Serna, Alejandro Sánchez‐Chardi, Verónica Nolan, Olivia Cano‐Garrido, Isolda Casanova, Ugutz Unzueta, Esther Vazquez, Ramon Mangues, Antonio Villaverde

**Affiliations:** ^1^ Institut de Biotecnologia i de Biomedicina Universitat Autònoma de Barcelona Bellaterra 08193 Barcelona Spain; ^2^ Instituto de Investigaciones Biológicas y Tecnológicas (IIBYT) (CONICET‐Universidad Nacional de Córdoba) ICTA & Cátedra de Química Biológica Departamento de Química FCEFyN, UNC. Av. Velez Sarsfield 1611 X 5016GCA Córdoba Argentina; ^3^ Departament de Genètica i de Microbiologia Universitat Autònoma de Barcelona Bellaterra 08193 Barcelona Spain; ^4^ CIBER de Bioingeniería Biomateriales y Nanomedicina (CIBER‐BBN) 28029 Madrid Spain; ^5^ Biomedical Research Institute Sant Pau (IIB‐Sant Pau) and Josep Carreras Research Institute Hospital de la Santa Creu i Sant Pau 08041 Barcelona Spain; ^6^ Servei de Microscòpia Universitat Autònoma de Barcelona Bellaterra 08193 Barcelona Spain; ^7^ Present address: Nanoligent SL Edifici Eureka Universitat Autònoma de Barcelona Bellaterra 08193 Barcelona Spain

**Keywords:** biomimetic materials, cancer, drug release, microparticles, recombinant proteins

## Abstract

Bacterial inclusion bodies (IBs) are mechanically stable protein particles in the microscale, which behave as robust, slow‐protein‐releasing amyloids. Upon exposure to cultured cells or upon subcutaneous or intratumor injection, these protein materials secrete functional IB polypeptides, functionally mimicking the endocrine release of peptide hormones from secretory amyloid granules. Being appealing as delivery systems for prolonged protein drug release, the development of IBs toward clinical applications is, however, severely constrained by their bacterial origin and by the undefined and protein‐to‐protein, batch‐to‐batch variable composition. In this context, the de novo fabrication of artificial IBs (ArtIBs) by simple, cell‐free physicochemical methods, using pure components at defined amounts is proposed here. By this, the resulting functional protein microparticles are intriguing, chemically defined biomimetic materials that replicate relevant functionalities of natural IBs, including mammalian cell penetration and local or remote release of functional ArtIB‐forming protein. In default of severe regulatory issues, the concept of ArtIBs is proposed as a novel exploitable category of biomaterials for biotechnological and biomedical applications, resulting from simple fabrication and envisaging soft developmental routes to clinics.

## Graphical abstract

Functional microscale protein granules can be obtained by simple physicochemical methods. These materials successfully mimic appealing properties of bacterial inclusion bodies, including amyloidal architecture, biological activity, and the release functional protein in vitro and in vivo. The applicability of these novel materials in biotechnology and in biomedicine is fully supported by their synthetic nature and defined chemical composition.

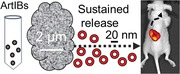

Bacterial inclusion bodies (IBs) are water‐insoluble proteinaceous inclusions generated in the cytoplasm of recombinant bacteria,^1^, ^2^ stabilized by an amyloid fibril architecture that confers mechanical robustness.^3^ They are formed by the transgene protein product plus a diversity of residual macromolecules from bacterial cells, including nucleic acids, carbohydrates, proteins, and cell wall components. Their morphology is defined by mechanical limitations imposed by the bacterial cell wall, usually at the edge of sub‐micrometer and micrometer sizes. The biological activity associated to IB proteins,^4^ together with the mechanical stability and high porosity of these protein particles has pushed to re‐evaluate them as unconventional functional materials with a wide spectrum of applications in biotechnology and biomedicine.^5^ Any functional polypeptide suitable for production in bacteria can be engineered to be packaged in form of IBs.^6^ This is because IBs show a complex structure with a dual organization of the forming polypeptide chains; while around 40% of the IB protein generates a mechanically stable fibril network, the remaining fraction represents functional or quasi‐functional species embedded in such stable structure.^7^ This protein population is properly folded, nearly soluble, releasable under physiological conditions, and responsible for the biological activity of IBs.^1^ As self‐immobilized catalysts, enzyme‐based IBs show high level of operational stability and reusability.^8^ In tissue engineering, they have been adapted as nontoxic topologies that provide a combination of mechanical and biological stimuli for controlled cell proliferation.^9^ In a different context, IBs have been tailored as unexpected drug delivery systems or “nanopills,”^10^ that mimic the functionalities of the secretory granules of the mammalian endocrine system^11^ for the intracellular, local or remote delivery of functional (either untargeted or receptor‐targeted) IB protein.^12^ Therefore, IBs, as functional biomaterials, show promise in protein replacement therapies and in any clinical uses aimed to systemic, local or precision protein delivery.

The enormous clinical potential of bacterial IBs is, however, darkened by their heterogeneous and undefined composition.^6^ Trapping an indeterminate catalogue of bacterial cell materials, clinically oriented IBs would hardly overcome the severe regulatory constraints imposed by medicament agencies. In this context, we wondered whether chemically pure IB mimetic particles (artificial IBs, ArtIBs) could be fabricated in vitro. This would imply the production of particulate microscale protein materials, made of pure and chemically controlled components, which would replicate those functionalities of natural IBs that are relevant to protein delivery. These properties are mechanical stability, absence of intrinsic cytotoxicity, mammalian cell penetrability, and the ability to release functional IB protein upon physiological conditions. Pure protein composition would be added to such functionally rich profile.

To explore the fabrication of synthetic IBs, common laboratory enzymes that form functional IBs during recombinant production, namely, β‐galactosidase (β‐Gal) and alkaline phosphatase (AP),^13^ were selected as models for two alternative approaches to ArtIB fabrication. In one (**Figure** **1**a), soluble protein was salted out plus thermally aggregated to generate amyloidal networks, further used as IB‐like seeds to recruit and entrap homologous soluble protein versions. This multiple step procedure (ms), was developed to imitate the dual, sponge‐like networks in natural IBs.^7^ Lipids, common component of bacterial IBs were also incorporated. In a simpler single‐step (ss) approach, divalent cations (Zn, in form of ZnCl_2_), involved in amyloid formation^14^ and generically, in protein–protein contacts,^15^ were added to a protein solution (Figure 1a). The application of these procedures resulted in mechanically stable, discrete, and moderately disperse protein particles sizing around 1–2 µm (AP) and 2–6 µm (β‐Gal) (Figure 1b,c), whose surface rugosity, size variability, and amorphous appearance remembered those of natural IBs (Figure 1b). Both enzymes, in this packaged form, were enzymatically active (Figure 1d). On the other hand, cross‐β‐sheet amyloid like structure (ALS) was detected by attenuated total reflectance (ATR) in AP ArtIBs at proportions (31–37%, ss and ms, respectively) matching those found in IBs.^7^

**Figure 1:**
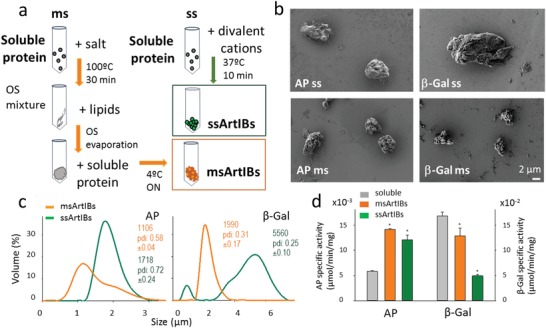
Fabrication of ArtIBs. a) Multiple (ms) and single (ss) step procedures for ArtIB fabrication from soluble pure protein are summarized, indicating the main operational steps (arrows). OS is organic solvent. Precise details can be found in the Experimental Section. Final products are framed. b) Representative field‐emission scanning electron microscopy (FESEM) images of AP and β‐Gal ArtIBs. Magnifications are equivalent in all images. c) Dynamic light scattering (DLS) size analyses of ArtIBs, indicating the mode (in nm) and the polydispersion index (pdi). d) Specific activity of both AP and β‐Gal ArtIBs, compared to that of commercial soluble protein counterpart. Asterisks indicate statistically different from the specific activity of the soluble protein (*p* < 0.001, Holme–Sidak test).

Further, we constructed new ArtIBs (**Figure** **2**a) formed by the self‐assembling modular proteins T22‐GFP‐H6^16^ and T22‐PE24‐H6,^17^ that are targeted to the cell‐surface cytokine receptor CXCR4^18^ through the N‐terminal tumor homing peptide T22.^19^ The size of these materials was smaller than that of those formed by the previously tested enzymes (Figure 1), a fact that, considering potential medical applications, could generally favor molecular and cell interactivity and protein release, by increasing the surface/volume ratio. When exposed to cultured CXCR4^+^ Hela cells, T22‐GFP‐H6 ArtIBs internalized very efficiently as in the case of IB‐based nanopills,^10^ by a CXCR4‐dependent route that is inhibited by the CXCR4 antagonist AMD3100^20^ (Figure 2b). Cell viability was not affected by T22‐GFP‐H6 ArtIBs (Figure 2c), but it was instead dramatically compromised, in a CXCR4‐dependent fashion, by the *Pseudomonas aeruginosa* exotoxin (PE24) contained in T22‐PE24‐H6 ArtIBs. As in the case of IBs, ArtIBs steadily released a fraction of the forming protein in soluble form when incubated in physiological buffer, at least for 7 days (Figure 2d). T22‐GFP‐H6 solubilized in vitro from ArtIBs was fluorescent (1039.83 AU mg^−1^), it assembled as 13 nm nanoparticles indistinguishable in size from soluble T22‐GFP‐H6 (Figure 2e), and this material was equally able to penetrate cultured HeLa cells in a CXCR4‐dependent way (Figure 2f). This fact unveiled a potential of ArtIBs as chemically homogenous protein reservoirs for prolonged in vivo delivery of tumor‐targeted, nanostructured protein drugs.

**Figure 2:**
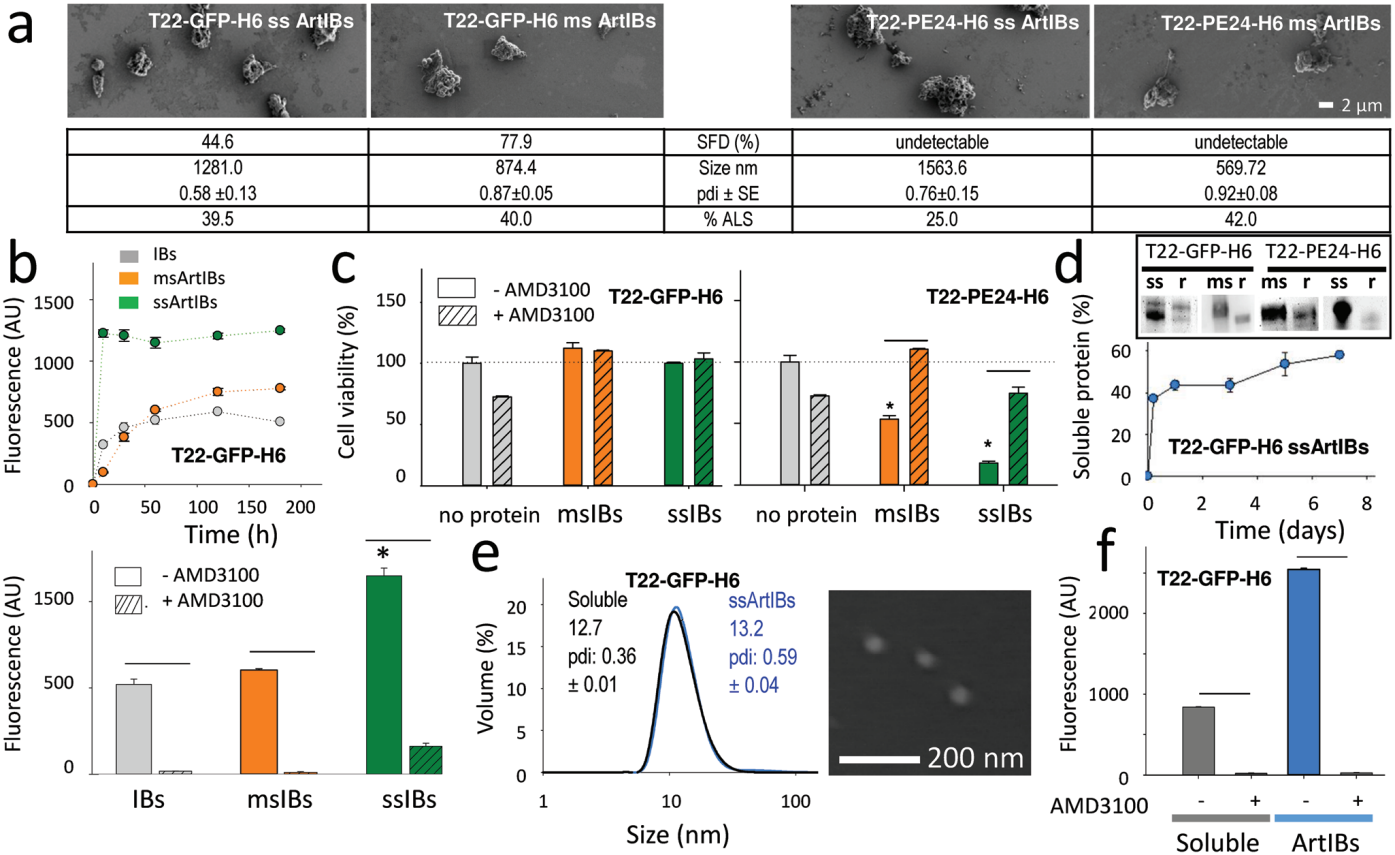
Characterization of ArtIBs formed by modular proteins. a) FESEM images of CXCR4‐targeted ArtIBs, all recorded at the same magnification. At the bottom of each image, specific fluorescence decay (SFD), hydrodynamic size peak (pdi ± s.e.m.) and percentage of ALS are shown. b) Internalization of T22‐GFP‐H6 ArtIBs in cultured HeLa cells, recorded at different times after exposure through intracellular green fluorescent protein (GFP)fluorescence (top). Bottom: AMD3100‐mediated inhibition of ArtIB internalization. c) Viability of cultured HeLa cells upon 96 h of T22‐GFP‐H6 and T22‐PE24‐H6 ArtIB exposure in presence or absence of AMD3100. d) Stain‐free protein detection of released soluble protein (r) from ArtIBs, 7 days after incubation in physiological buffer. In the plot, kinetics of soluble protein release from T22‐GFP‐H6 ssArtIBs. e) Hydrodynamic mode size peak of T22‐GFP‐H6 nanoparticles released from ssArtIBs, compared to equivalent soluble nanoparticles after purification from recombinant bacteria (those used for ArtIB fabrication). In the inset, an FESEM image of those nanoparticles released from ArtIBs. f) AMD3100‐mediated inhibition of HeLa cell internalization of recombinant soluble and ArtIBs‐released nanoparticles. Symbols indicate significant differences to the control (*, *p* < 0.05, Tukey test) and between samples with or without AMD3100 (^—^, *p* < 0.05, two tail, *t*‐test).

In this context, different categories of T22‐GFP‐H6 ArtIBs were implanted subcutaneously (SC) in a CXCR4^+^ colorectal cancer mouse model, releasing fluorescent material from the implantation point, followed by selective uptake by a remote CXCR4^+^ tumor, with specific kinetics for each ArtIB type. A preliminary screening of T22‐GFP‐H6 msArtIBs and T22‐GFP‐H6 ssArtIBs (Zn^2+^, at 100:1 ratio of zinc to protein) showed slow release and negligible or a small amount of material accumulated in the tumor by 21 days (**Figure** **3**a). Lowering the proportion of Zn^2+^ (50:1) or using alternative cations to induce ssArtIBs formation, improved protein release and tumor uptake. In particular, ss ArtIBs formed by Ca^+2^ were more efficient than ArtIBs (Zn^+2^ 50:1) in maintaining a faster and progressive protein release from the SC injection site leading to a higher accumulation and longer residence time (starting at day 3 and at least until day 10) in the remote CXCR4^+^ tumor (Figure 3b). Further, T22‐PE24‐H6 ArtIBs Ca^2+^, containing the CXCR4‐targeted cytotoxic polypeptide PE, induced an (*p* = 0.083) inhibition of tumor growth (*n* = 3, 1.0 ± 0.2 × 10^8^) stronger than T22‐GFP‐H6 Ca^2+^ ArtIBs (*n* = 3, 1.5 ± 0.7 × 10^8^), as compared to the control buffer‐treated group (*n* = 2, 2.6 ± 1.0 × 10^8^) (Figure 3c). This fact occurred in absence of systemic toxicity (lack of histopathological alterations in hematoxylin and eosin (H&E)‐stained liver and kidney at the end of the experiment, not shown). These observations fully confirmed both the secretion‐like prolonged protein release and the precise cell targeting of functional materials through the blood stream, from a remote location.

**Figure 3:**
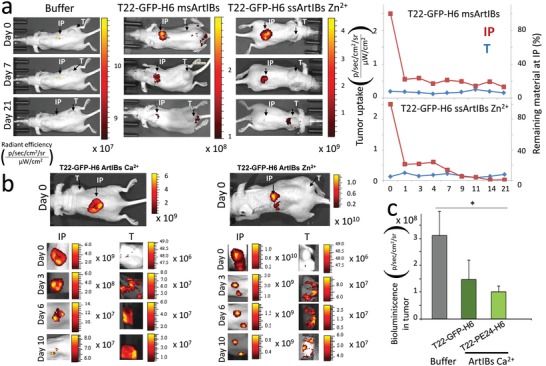
ArtIBs material release, tumor uptake and antitumor activity in a CXCR4^+^ colorectal cancer model. a) Preliminary screening of released material and tumor uptake after subcutaneous implantation of T22‐GFP‐H6 msArtIBs, T22‐GFP‐H6 ssArtIBs (Zn^2+^ 100:1) or PBS. b) Representative FLI images obtained at the injection point (IP) and at the remote tumor (T), along time (day 0, 3, 6, and 10) after T22‐GFP‐H6 ssArtIBs Ca^2+^, T22‐GFP‐H6 ssArtIBs Zn^2+^ (1:50) or buffer SC administration. c) Antitumor effect, measured as bioluminiscence emission by cancer cells along time, in the CXCR4^+^ SW1417‐luci tumor model, after SC injection of 1 mg dose per mouse of T22‐GFP‐H6 Ca^2+^ArtIBs, T22‐PE24‐H6 Ca^2+^ ArtIBs or control PBS (*, *p* < 0.05, Tukey test). Fluorescence in (a) and (b), or bioluminescence in (c) intensity were measured using IVIS Spectrum and expressed as x‐ ± SE of average radiant efficiency.

In summary, ArtIBs can be fabricated in vitro as a new type of biomimetic material, from pure protein and by simple physicochemical methods. These protein particles reproduce IB properties that are relevant to potential uses in biomedicine, especially protein release, but their potential use would fully prevent the immunotoxic reactions potentially associated to the administration of IBs, that contain bacterial debries at variable but significant proportions. On the other hand, since protein drugs used in clinics have a human origin,^21^ their administration in an ArtIBs format is not expected to pose significant immune concerns, or to enhance any putative immune reaction over those associated to the repeated, conventional administration regimens of soluble protein drugs (such as insulin, interferons, and many others). In particular, the simpler ss fabrication method allows engineering the strength of protein–protein interactions in the material by means of the stoichiometric control of metal or nonmetal divalent cations. In contrast to other excellent and biocompatible materials developed as micro‐ or nanoparticles for the slow and sustained drug release,^22^ such as those based on PLA or PLGA,^23^ ArtIBs are chemically homogeneous and show no chemical distinction between carrier and cargo, thus acting as self‐contained, self‐released drug materials. Then, the protein drug itself acts, in addition, as a scaffold material, what results in intriguing, totally novel and chemically homogenous drug delivery systems with simpler fabrication processes as opposite to hybrid platforms. ArtIBs might not only replace IBs as functional protein reservoirs and offer homogeneous materials for drug‐oriented development, but they will enable, in addition, the packaging of glycosylated proteins of mammalian cell origin as IB‐like materials. Since these proteins would be never produced in bacteria in functional forms, ArtIBs will then expand, as a universal platform, the catalogue of enzymes or protein drugs that could be formulated as pure microscale biocatalysts or as secretory protein granules.

## Experimental Section

*Fabrication of ArtIBs*: To produce msArtIBs, 1 mg of pure soluble protein was denatured and concomitantly precipitated by heating at 100 °C in NaCl_2_ (500 × 10^−3^ m), ZnCl_2_ (26.4 × 10^−3^ m), and MgCl_2_ (18.4 × 10^−3^ m) in distilled H_2_O. The precipitate was centrifuged at 15 000 × *g* for 15 min at 4 °C, isolated from the soluble fraction and resuspended with 1 mg of phosphatidylcholine with chloroform/methanol 2:1 v/v in a final volume of 300 µL. The excess of organic solvent (OS) was removed by a continuous N_2_ flow inducing the formation of a protein–lipid film phase that acted as scaffold. The scaffold was afterwards resuspended in 1 mg mL^−1^ of previous soluble protein diluted in phosphate buffered saline (PBS) at 4 °C overnight. Finally, the sample was centrifuged, and soluble fraction discarded. The manufacturing of ssArtIBs was approached by diluting pure soluble protein in distilled H_2_O at a final concentration of 2 mg mL^−1^ and final volume of 200 µL. Protein samples (0.196 × 10^−3^ m) were subsequently mixed with ZnCl_2_, at a 100:1 ratio of zinc to protein. After 10 min of incubation at room temperature, samples were centrifuged at 15 000 × *g* for 15 min and soluble fraction discarded to obtain the final product. Alternatively, zinc at a ratio 50:1 and calcium at a ratio 300:1 (in form of CaCl_2_) were used for the in vivo experimental. β‐Gal [EC3.2.1.23] and AP [EC3.1.3.1], both from *Escherichia coli*, were purchased from Sigma‐Aldrich. T22‐GFP‐H6 and T22‐PE24‐H6 were produced as recombinant proteins and purified by single step chromatography as reported.^17^

*Determination of Enzymatic Activity*: Between 3.7 and 21.3 ng of pure soluble β‐Gal protein or β‐Gal ArtIBs were mixed with 5 × 10^−3^ m of *ortho*‐nitrophenylgalactopiranoside in a final volume of 500 µL of PBS. The mixture was incubated for 15 min at 37 °C, the reaction stopped by adding 200 µL of Na_2_CO_3_ (2.8 m) and the product amount determined by measuring absorbance at 420 nm (ε_420_ = 4530 m ^−1^ cm^−1^) in a UV–vis spectrophotometer (Ultrospec 1000E, Pharmacia Biotech). On the other hand, between 3.9 and 92 ng of pure soluble AP protein or AP ArtIBs were mixed with 20 × 10^−3^ m of *para*‐nitrophenylphosphate (pNPP) in a final volume of 500 µL of PBS. The mixture was incubated for 15 min at 37 °C, the reaction stopped by adding 200 µL of NaOH (1 m) and activity determined by measuring p‐nitrophenyl phosphate absorbance at 405 nm (ε_405_ = 18 000 m ^−1^ cm^−1^) in a UV–vis spectrophotometer (Ultrospec 1000E, Pharmacia Biotech).

*Determination of Specific Fluorescence*: Pure soluble T22‐GFP‐H6 or ArtIB versions were diluted in PBS at concentrations ranging from 0.2 to 1 mg mL^−1^. The excitation wavelength (λ_ex_) was set at 488 nm and the emission (λ_em_) at 510 nm, meanwhile the excitation slit was set at 2.5 nm and the emission slit at 5 nm. Fluorescence was measured in a Cary Eclipse Fluorescence Spectrophotometer (Agilent Technologies) by using a quartz cell with a 10 mm path of light. The intrinsic fluorescence of each sample was then represented referred to protein concentration, defining the SFD mathematically represented as a slope. The % of SFD (%SFD) represents the relationship of the parameter with the SFD of soluble T22‐GFP‐H6 protein.

*Size Distribution Analysis*: Volume size distribution of all nanostructures was determined at 633 nm and 25 °C in a Zetasizer Nano ZS (Malvern Instruments Limited) by using ZEN2112 3 mm quartz batch cuvettes. Protein samples dissolved in PBS from 0.2 to 1 mg mL^−1^ were measured in triplicate and mode size peak and polydispersion index (pdi ± s.e.m.) obtained.

*Electron Microscopy*: Ultrastructural morphometry (size and shape) of ArtIBs was characterized at nearly native state with field emission scanning electron microscopy (FESEM). Drops of 20 µL of each sample diluted at 0.3 mg mL^−1^ in their respective buffers were directly deposited on silicon wafers (Ted Pella Inc.) for 30 s and immediately observed without coating with an FESEM Zeiss Merlin (Zeiss) operating at 1 kV and equipped with a high resolution secondary electron detector. Representative images of a general fields and nanoparticle detail were captured at magnifications ranging between 5500× and 8500× and a working distance of 3.5 mm.

*Attenuated Total Reflectance*: The most suitable concentration of ArtIBs was placed and dried with a continuous N_2_ flow on spectroscopic crystal surfaces. Total reflectance spectroscopy was detected 15 times as spectra by using a scan rate of 50 cm^−1^ min^−1^ and a nominal resolution of 2 cm^−1^ in a Tensor 27 Bruker spectrometer coupled to a Specac Golden Gate ATR accessory. All measurements were performed at 25 °C, the absorbance obtained was corrected against the background and the PBS buffer signal was subtracted. Fourier deconvolution of the spectra and the second derivative allow the identification of the different band components. Fitting of the components to the original (not deconvolved) spectrum was essentially performed according to a described procedure.^24^ Peak height, band width, and peak position of the components were allowed to vary one at a time in this order. A Gaussian shape was assumed.

*Cell Culture*: CXCR4^+^ cervical cancer cell lines (HeLa ATCC‐CCL‐2) were used to study the performance of ArtIBs in vitro. Cells were routinely cultured in Eagle's minimum essential medium (Gibco), supplemented with 10% fetal bovine serum (FBS, Gibco) and incubated in a humidified atmosphere at 37 °C and 5% of CO_2_.

*Protein Internalization*: HeLa CXCR4^+^ cells were cultured in 24‐well plates in MEM Alpha 1× GlutaMAX medium (Gibco) supplemented with foetal bovine serum (FBS) at 37 °C in a 5% CO_2_ humidified atmosphere until 70% of confluence was reached. The medium was then exchanged for serum free OptiPro medium (Gibco) before the addition of the protein. Protein uptake was determined at different times ranging from 10 min to 24 h at a final concentration of 2.5 µg. Cells were detached, and external hooked protein removed by Trypsin‐EDTA (Gibco) at 1 mg mL^−1^ exposure for 15 min at 37 °C. Intracellular protein fluorescence was detected by flow cytometry using a FACS‐Canto system (Becton Dickinson) with an air‐cooled argon ion laser (15 mW) exciting at 488 nm and a D detector (530/30 nm as band pass filter). In addition, the internalization specificity through CXCR4 receptor was tested by exposing cells to the CXCR4 antagonist AMD3100^25^ 1 h prior protein incubation at (protein/AMD3100) 1:10 ratio.

*Cell Viability*: HeLa (ATCC‐CCL‐2) cell line was cultured in opaque‐walled 96‐well plates at a final concentration of 6000 cells per well for 24 h. MEM Alpha GlutaMAX medium (Gibco) supplemented with FBS was used at 37 °C in a 5% CO_2_ humidified atmosphere, until 70% of confluence was reached. ArtIBs were incubated at 1 × 10^−6^ m for 96 h using MEM Alpha GlutaMAX medium (Gibco). Cell viability was measured by CellTiter‐Glo Luminescent Cell Viability Assay (Promega) in a Multilabel Plater Reader Victor3 (Perkin Elmer).

*Soluble Protein Release from Artificial IBs*: ArtIBs were resuspended in 1 mL of PBS 1× reaching a final concentration of 1 mg mL^−1^ and incubated at 37 °C without agitation. 100 µL were taken from each sample at different times ranging from 0 to 7 days and centrifuged for 15 min at 15 000 × *g* at 4 °C to isolate soluble and insoluble fractions. Soluble protein was then stain‐free detected by TGX (TGX FastCast Acrylamide Kit) and subsequently quantified by ImageLab software to determine the % of released protein.

*In Vivo Release of Fluorescent Material by Subcutaneously Implanted ArtIBs and Their Tumor Uptake*: Four‐week‐old female mice of the Swiss nude strain, in the 18–20 g body weight range (Charles River, L‐Abreslle, France), maintained in pathogen‐free conditions, were used for the in vivo experiments. All experimental procedures were approved by the Hospital de Sant Pau Animal Ethics Committee and performed according to European Council directives. To generate the CXCR4^+^ SW1417 CRC cancer model, a 10 mg aliquot of SW1417‐luci tumor tissue was obtained from donor animals and deposited in the anterior or posterior flank subcutis of the animals. When tumors reached ≈120–200 mm^3^ volume, animals were randomly allocated and implanted in the subcutis of the mouse lumbar region with a pellet of T22‐GFP‐H6 msArtIBs or T22‐GFP‐H6 ssArtIBs Zn^2+^ in a preliminary study, at a single dose injection of 1 mg per mouse, suspended in a 150 µL PBS buffer, whereas in a second study, T22‐GFP‐H6 Zn^2+^ ArtIBs or of T22‐GFP‐H6 Ca^2+^ ArtIBs were implanted at the same dose. Control buffer injection was used as a negative control. The ArtIBs IP was selected to position it as far away as possible from the tumor in the same mouse, being located either in the anterior or posterior flanks.

After ArtIBs pellet injection, the IVIS Spectrum equipment (PerkinElmer Inc.) was used to monitor the GFP‐emitted fluorescence by the SC implants in whole‐body mouse by registering immediately (0 h) and at specific time points (3, 6, and 10 days) after the administration to determine the fluorescence remaining in the subcutaneous ArtIBs implants, as well as the fluorescent material that reached the remote tumor along time, in each mouse. Fluorescent signal was digitalized, displayed as pseudocolor overlay, and expressed as radiant efficiency. The fluorescence intensity (FLI) ratio was calculated dividing the signal from the IBs‐treated mice by the FLI autofluorescent signal of buffer‐administered control mice either in the injection point or in the tumor.

*In Vivo Antitumor Activity of SC Implanted ArtIBs*: The CXCR4^+^ SW1417 CRC cancer model used to test antitumor activity was generated as described above. The expression of luciferase by cancer cells in this model allowed for the noninvasive follow‐up of tumor growth along time. A week before the deposition of the tumor aliquot in the mouse subcutis, mice were randomly allocated to be SC administered in the mouse lumbar region with 1 mg per mouse dose of T22‐GFP‐H6 Ca^2+^ ArtIBs or T22‐PE24‐H6 Ca^2+^ ArtIBs suspended in a 150 µL of PBS buffer or buffer‐treated control mice.

After IBs administration, mouse body weight was recorded, and bioluminescent image intensity in the tumor, measured using the IVIS Spectrum equipment (PerkinElmer Inc.), was digitalized and expressed as radiant efficiency. Tumor tissue, liver, and kidney were formalin‐fixed and paraffin‐embedded for histology. To that aim, four‐micrometer‐thick sections were stained with H&E, and analyzed for possible histological alterations by two independent observers. Representative images were taken using Cell^B software (Olympus Soft Imaging v. 3.3).

*Statistical Analysis*: All analyses were performed with SPSS versus version 11.0 (IBM) software. One‐way ANOVA and *t*‐tests were performed to assess differences in assays with a minimum *n* = 3. The Holme–Sidak test was applied for equal variance and Tukey or Mann Whitney U test for unequal variance (indicated in the figure legend). Two tail *t*‐test was also used for individual comparisons. Data were presented as means ± standard error of the mean (s.e.m). Differences between the protein samples were considered significant at *p* ≤ 0.05.

## Conflict of Interest

E.V., R.M., and A.V. are cofounders of NANOLIGENT, devoted to develop antitumoral drugs based on proteins. J.M.S., H.L‐L., P.A., E.V., R.M. and A.V. are co‐inventors in a patent covering the use of ArtIBs.
